# Uretero-neocystostomy: a retrospective comparison of open, laparoscopic and robotic techniques

**DOI:** 10.1186/s12894-023-01200-1

**Published:** 2023-03-07

**Authors:** Christian Ramesmayer, Maximilian Pallauf, Ricarda Gruber, Thomas Kunit, David Oswald, Lukas Lusuardi, Michael Mitterberger

**Affiliations:** 1 Department of Urology and Andrology, Paracelsus Medical University, Müllner Hauptstraße 48, 5020 Salzburg, Austria; 2 Department of Urology, Comprehensive Cancer Center, Medical University Vienna, Vienna, Austria; 3 Department of Urology and Andrology, Pyhrn-Eisenwurzen Klinikum Steyr, Steyr, Austria

**Keywords:** Lower ureteric obstruction, Ureteral reimplantation, Uretero-neocystostomy, Laparoscopy, Robotic

## Abstract

**Background:**

Uretero-neocystostomy (UNC) is the gold-standard for distal-ureter repair. Whether the surgery should be conducted minimally invasive (laparoscopic (LAP), robotic RAL)) or open remains unanswered by the literature.

**Methods:**

Retrospective analysis of surgical outcome of patients treated with UNC for distal ureteral stenosis (January 2012 - October 2021). Patient demographics, estimated blood loss (EBL), surgical technique, operative time, complications and length of hospital stay (LOS) were recorded. During the follow-up period, patient underwent renal ultrasound and kidney function tests. Success was defined as relieve of symptoms or no findings of obstruction needing urine drainage.

**Results:**

60 patients were included (9 RAL, 25 LAP, 26 open). The different cohorts were similar of age, gender, American Society of Anesthesiologists (ASA) score, body-mass index and history of prior treatment of the ureter. No intraoperative complications were detected in all groups. There was no conversion to open surgery in the RAL group, whereas one was found in the LAP arm. Six patients had a recurrent stricture, but with no significant difference between the cohorts. EBL was not different between the groups. LOS was significantly lower in the RAL + LAP group compared to open (7 vs. 13 days, p = 0.005) despite significantly longer operating times (186 vs. 125.5 min, p = 0.005).

**Conclusion:**

Minimal invasive UNC, especially RAL, is a feasible and safe surgical method and provides similar results in terms of success rates in comparison to open approach. A shorter LOS could be detected. Further prospective studies need to be done.

## Background

There are multiple causes for ureteral obstruction, of which ureter stones, iatrogenic injuries, and malignancies are the most frequent ones [1]. The definitive treatment for distal ureteral obstruction is ureteroneocystostomy (UNC). This surgery used to be performed with an open approach [2]. However, implementing new surgical techniques, such as the psoas hitch and the Boari-flap technique, made it possible to treat ureteral stenosis minimally invasive as well [3].

With the laparoscopic (LAP) approach promising success rates and improvements in terms of estimated blood loss (EBL), length of hospital stay (LOS) and convalescence time in comparison to the open technique were reported [4]. The robot-assisted laparoscopic (RAL) approach has become an attractive option in the last years for UNC with comparable success rates, as among other things steep learning curves due to facilitation of suturing during urological procedures were reported in several studies [5, 6]. Only a few case series comparing open with laparoscopic or robotic-assisted technique have been published so far [7–9]. As the number of publications is scarce, we made a retrospective comparison of all three modalities for UNC.

### Methods

An ethical approval was obtained from the local ethical committee to conduct our retrospective study. We retrospectively included all patients with an ureteral stenosis, who were treated with UNC either in an open, laparoscopic or robotic assisted technique at the Department of Urology and Andrology of the Paracelsus Medical University Salzburg, Austria from January 2012 until October 2021. The minimal invasive (RAL and LAP) group was compared with the open group. We included all etiologies for stenosis. Patients with insufficient data or loss of follow-up were excluded from the study. Data were collected from patient medical records.

We assessed the following information: Age, gender, laterality, American Society of Anesthesiologists (ASA) score, body-mass index (BMI), history of previous abdominal surgery, history of prior ureteral stricture treatment, stricture etiology, estimated blood loss (EBL, calculated by the difference of pre- and postoperative hemoglobin), pre- and postoperative serum creatinine, operative time, technique (pure ureteroneocystostomy, psoas hitch, Boari flap, distal ureterectomy), intraoperative complications and length of hospital stay (LOS). The ureteral procedure was based primarily on stricture length and on surgeon’s choice. Postoperative success was defined as relieve of symptoms or no radiological signs of obstruction needing drainage or reoperation. We also recorded any complications according to Clavien-Dindo system [10]. Major complications were stated as Clavien-Dindo 3a or higher.

#### Perioperative evaluation

We collected the following values of all patients who underwent a planned UNC: blood chemistry, blood count, urine analysis and culture. In case of scheduled operation all patients received cross-sectional imaging with at least one contrast-enhanced computed tomography scan. Patients who were treated initially with nephrostomy underwent antegrade ureterography to define stricture length. In case of no complete obstruction or an unclear function of the affected kidney a mercaptoacetyl triglycine (MAG) 3 scan with “Lasix” (Furosemide) was performed. Unscheduled patients due to emergency operation (e.g. intraoperative iatrogenic injury) were staged during the operation by the consultant urologist. Except for a few cases, all ureteral anastomoses were stented with a pigtail ureteral stent. An anastomotic drain was placed in all cases. Postoperatively, the perianastomotic drain was removed when its output was low (< 50ml/day) and the creatinine levels showed no urinary leakage. After a normal cystography the urethral catheter was removed 5–10 days postoperative depending on the surgeon’s individual choice. The ureteral stent was removed about 6 weeks after the operation. Two months after stent removal the patients underwent renal ultrasound and in unclear cases nuclear scans were done. The next follow-up was done 6-months later. Any further evaluation was left to the treating urologist’s discretion.

#### Surgical technique

The open and LAP procedures for UNC were well described by former authors and performed as mentioned in papers like Rassweiler et al. [11]. The reimplantation technique was decided by the treating physician during the surgical procedure. The simple UNC-only technique (no opening of Retzius space and bladder mobilization due to small stricture length) was performed if a tension-free direct anastomosis was possible. If the stricture length made a tension-free direct anastomosis between bladder and ureter impossible, the psoas-hitch technique was performed by fixation of the urinary bladder on the tendon of the ipsilateral iliopsoas muscle. The Boari-flap procedure was the adequate surgical technique for longer strictures. All anastomoses were performed refluxive and extravesical. Stricture length was not routinely and only quite inaccurately assessed. As a result, we cannot state if the stricture length affected the implantation technique.

RAL had been conducted as follows: All RAL UNC were performed using the “da Vinci X Surgical System” (Intuitive Surgical, Sunnyvale, CA). The patient was placed into the moderate Trendelenburg position. All procedures were performed transperitoneal with a 12-mm camera port supraumbilical, three 8-mm trocars and one 12-mm port for the assistance. After achievement of a pneumoperitoneum and identification of the iliac vessels, an ureterolysis was performed proximal and distal of the stricture. The stricture was then resected to rule out malignancy. The ureteral reconstruction was based on surgeon’s choice. In all robotic cases, a Double-J stent was placed in the ureter. After completion of the anastomosis, we filled the bladder with 250 ml of saline to rule out any leakage.

### Statistical analysis

All data of continuous variables were checked for normal distribution (test of normality: Kolmogorov-Smirnov with Lilliefors significance correction, type I error = 10%). Continuous variables with normally distributed data were compared between the subgroups (open vs. robotic and lap) by the t-test for independent samples. For comparisons of continuous variables without normally distributed data and of the one variable measured on ordinal scales (ASA score) the exact Mann-Whitney U test was used. Dichotomous variables were compared by the Fisher’s exact test, the other categorical variables by the exact chi-square test.

The type I error was not adjusted for multiple testing. Therefore, the results of inferential statistics are descriptive only. Statistical analyses were performed using the open-source R statistical software package, version 4.1.2 (The R Foundation for Statistical Computing, Vienna, Austria).

## Results

We included a total of 60 patients (31 male) with a median age of 55.5 (interquartile range [IQR] 44.7–72.0) years. Nine patients underwent the RAL approach, 25 the LAP approach and 26 patients were treated with the open technique. The median follow up was 110 weeks. Patients were similar of age, laterality, BMI and ASA score. Preoperative data is summarized in Table 1.

**Table 1:** Demographic characteristics

	RAL + LAP	Open	P-value
**Number of cases**	34	26	
**Age, median - yr**	50 (41–70)	60 (53–74)	**0.048**
**Body mass index**	24.6 (22.5–28)	24.9 (21.1–27.9)	0.654
**Male sex- no. (%)**	17 (50)	14 (53.8)	0.800
**Prior abdominal surgery – no. (%)**	16 (48.5)	21 (80.8)	**0.015**
**ASA score – no (%)**			**0.027**
1	5 (14.7)	1 (3.8)	
2	22 (64.7)	13 (50.0)	
3	7 (20.6)	12 (46.2)	
**Side – no (%)**			**0.045**
Left	15 (44.1)	12 (46.2)	
Right	19 (55.9)	10 (38.5)	
Bilateral	0	4 (15.4)	
**Prior treatment**			**0.013**
None	8 (23.5)	12 (46.2)	
DJ-Stent	21 (61.8)	6 (23.1)	
PCN	5 (14.7)	7 (26.9)	
Other definitive	0	1 (3.8)	
Other non-definitive	0	0	
**Etiology**			0.666
Stricture	15 (44.1)	7 (26.9)	
Malignancy	3 (8.8)	4 (15.4)	
Iatrogenic	10 (29.4)	9 (34.6)	
Stone	2 (5.9)	1 (3.8)	
Other	4 (11.8)	5 (19.2)	

RAL = robotic assisted laparoscopic; LAP = laparoscopic; ASA = American Society of Anesthesiologists; PCN = Percutaneous nephrostomy

Operative data is summarized in Table 2. In the LAP and RAL cohort the psoas hitch technique was preferred. Operating time was significantly shorter in the open cohort (125.5 (105–180) vs. 186 min (140–224), p = 0.005).

The estimated blood loss, calculated by the difference of pre- and postoperative hemoglobin levels, was comparable between the groups (0.4 RAL + LAP vs. 0.95 open). No intraoperative complications or challenging difficulties were reported in all groups. One conversion to open surgery was found in the LAP group due to previous abdominal operations and therefore resulting visceral adhesions. The patient was finally treated with psoas-hitch technique.

**Table 2:** Perioperative data

	RAL + LAP	Open	p-value
Number of cases	34	26	
Operative time - min	186 (140–224)	125.5 (105–180)	**0.005**
Conversion to open technique	1 (2.9)	n/a	> 0.999
Technique – no (%)			**0.021**
UNC - only	5 (14.7)	13 (50.0)	
UNC - psoas	22 (64.7)	11 (42.3)	
UNC - boari	4 (11.8)	1 (3.8)	
UNC – distal ureterectomy	3 (8.8)	1 (3.8)	
EBL - Δ Hemoglobin	0.4 (0.0–1.4)	0.95 (0.5–1.9)	0.592

RAL = robotic assisted laparoscopic; LAP = laparoscopic; ASA = American Society of Anesthesiologists; UNC = Ureteroneocystostomy; EBL = Estimated blood loss

In Table 3 the postoperative data is summarized. A significant difference in LOS could be detected in the minimal-invasive-arm compared to open (p = 0.005). The RAL and LAP group had a median LOS of 9 (7–12), whereas the open cohort 13 (9–19) days. No complaints or urinary tract infections were reported postoperatively. Major complications (Clavien Dindo 3a or greater) were lower in the minimal invasive group with statistical significance (p = 0.032). One patient in the RAL group underwent another operation due to urinoma in the area of the implantation of the ureter into the bladder. Three sutures were placed in the bladder and the patient recovered well. An abdominal abscess measuring 5 × 5 cm in iliopsoas muscle made an operative revision necessary in one patient who was treated with LAP technique: The patient was treated with an open abscess drainage and recovered well. Seven patients (26.9%) in the open cohort required surgical consult. One patient in the open cohort died three days after the operation due to pulmonary embolism. The 90-day readmission rate of 0% (RAL + LAP) and 16% (open) showed statistical significance (p = 0.028). The change in creatinine level did not differ between the groups. The failure rate of 5.9% (RAL + LAP) and 15.4% (open) showed no significant changes in statistical analysis.

**Table 3:** Postoperative data

	RAL + LAP	Open	p-value
Number of cases	34	26	
LOS, median - days	9 (7–12)	13 (9–19)	**0.005**
Δ Creatinine, median	0.08 (0.0-0.15)	0.02 (0.00-0.14)	0.059
Stent duration, median - days	42 (28–42)	28 (18–42)	0.367
Foley-catheter duration, median -days	7 (5–10)	7 (6–10)	0.941
90-day readmission, no. (%)	0	4 (16.0)	**0.028**
Major complication, no. (%)	2 (5.9)	7 (26.9)	**0.032**
Failure, no. (%)	2 (5.9)	4 (15.4)	0.388

RAL = robotic assisted laparoscopic; LAP = laparoscopic; LOS = Length of hospital stay

## Discussion

We retrospectively compared the surgical outcome of robotic- and laparoscopic with open ureteroneocystostomy. Both groups showed comparable demographics. However, the RAL group included the smallest number of patients (9 vs. 25 vs. 26), representing an operative procedure, that has been implemented only a few years ago. These differences in patient numbers in subgroup size can be found in other publications as well, even if only two operative techniques were compared [8, 9, 12]. Despite this drawback concerning cohort size, we could demonstrate that the RAL-technique is safe and feasible.

The surgery time varied between the groups, giving an advantage for open surgery. However, surgery time is highly dependent on the ureteroneocystostomy technique. The simple UNC-only technique was most frequently used within the open surgery group. In contrast, the more time-consuming psoas-hitch technique was preferred for RAL and LAP. The challenging and time-consuming procedure of intracorporal suturing in laparoscopy is an often-postulated reason for longer surgery time in LAP. The RAL UNC with its advantage of three-dimensional visualization and seven degrees of freedom at the instrument wrist has helped to master the difficulties that are known in LAP technique. In our open cohort the operating time was significantly shorter with a median time of 125.5 min compared to the minimal invasive arm with 186 min (p = 0.005). When reviewing literature, difference in operating time is rarely reported. Most groups reported no differences in operating time [7, 12, 13]. In contrast, a significant longer operating time in the RAL group compared to the open was shown by Isac et al. (279 vs. 200 min, p < 0.01) [8]. Elsamra et al. detected no differences in operative time (236 vs. 235 vs. 257 min, p = 0.123) in all three groups (RAL, LAP, open) [9].

Similar to surgery time, there was a trend for a shorter LOS for the minimally invasive techniques. In literature, minimal invasive surgery results in shorter LOS compared to open. In summary, the publications mainly revealed significant lower LOS, especially in robotic but also in minimal invasive techniques [8, 9]. Interestingly, Baldie et al. showed similarity in terms of LOS in the LAP and RAL technique in benign strictures [7]. These published results are consistent with our data: LOS is lower in the minimal invasive arm compared to the open technique in benign and malignant cases (9 vs. 13 days, p = 0.005).

Our study revealed a comparable surgical outcome and safety for all techniques. Open surgery, RAL and LAP had similar EBL, complication rates, 90-day readmission rates, and failure rates with some advantages for the minimal invasive techniques. When comparing the literature, Patil et al. [12] were the first who demonstrated lower EBL and similar success rates in the RAL group compared to open and LAP cohorts. Kozinn and colleagues confirmed these results in benign diseases [13]. Interestingly, Baldie et al. showed similarity in terms of success and EBL in the minimal invasive techniques in benign strictures [7]. In 2013 Isac et al. presented a comprehensive comparison of 25 RAL versus 41 open UNC [8]. The by then largest published study comparing these two modalities reported lower EBL (100 vs. 150 ml, p < 0.01) in the RAL arm with comparable outcome. To our knowledge, there has been only one study which compared all three modalities in benign and malignant diseases so far (20 RAL, 85 LAP and 25 open procedures) [9]. Not surprisingly, the published data from Elsamra et al. documented significant lower EBL (100 vs. 150 vs. 300 ml, p < 0.01) in minimal invasive procedures but no significant difference in success rates. In summary, literature revealed significant lower EBL and comparable success rates, especially in robotic but also in minimal invasive techniques. Our data are consistent with these publications. Even though many groups were able to detect differences in EBL, our study could not show a difference in EBL due to poorly recordings in the anesthesiological protocol in our clinic.

At our institution, we started performing robotic surgery in 2019, in particular RAL UNC. We have gained significant surgical experience with robotic urological procedures since then by performing at least 200 per year. Even though these nine patients were our first patients treated with RAL UNC, we had comparable results together with LAP compared to the open group. The RAL technique seems to have a shorter learning curve for the surgeon compared to LAP. This hypothesis is confirmed in publications regarding other operative urological procedures [14]. Moreover, due to dexterity and facilitation of suturing, more urologists are able to perform UNC minimally invasive robotic assisted, without the formerly essential experience in pure laparoscopy.

There were some limitations to our study. Next to the retrospective design itself with a concomitant selection bias, the small sample size in the RAL arm and the heterogeneity in the groups with the inclusion of malignancies as etiology are mentionable limitations. Due to the few robotic cases, we combined the RAL with the LAP technique and compared it with open. Stricture length was not routinely assessed and therefore we cannot state if the length affected the implantation technique. Furthermore, the EBL was calculated with pre- and postoperative hemoglobin difference and not with the intraoperative measured blood loss. In spite of that, this is the second largest retrospective study comparing all three modalities for UNC.

## Conclusion

Minimal invasive UNC, especially RAL, is a feasible alternative for the definitive treatment of distal ureteral stenosis compared to the open approach. Our retrospective cohort study could demonstrate its safety and a short learning curve. Bigger case series, ideally in a prospective design, need to be done in the future to evaluate the effectiveness of RAL UNC.

## Data Availability

The datasets generated and/or analysed during the current study are not publicly available due to no open access to Paracelsus Medical University medical records data base, but are available from the corresponding author on reasonable request.

## List of abbreviations

UNC: Ureteroneocystostomy
LAP: Laparoscopic
EBL: Estimated blood loss
LOS: Length of hospital stay
RAL: Robotic-assisted laparoscopic
ASA: Anesthesiologists score
BMI: Body-mass index
MAG: Mercaptoacetyl triglycine
IQR: Interquartile range
PCN: Percutaneous nephrostomy
